# Intratumoral collagen signatures predict clinical outcomes in feline mammary carcinoma

**DOI:** 10.1371/journal.pone.0236516

**Published:** 2020-08-10

**Authors:** Suzanne Rosen, Becky K. Brisson, Amy C. Durham, Clare M. Munroe, Conor J. McNeill, Darko Stefanovski, Karin U. Sørenmo, Susan W. Volk

**Affiliations:** 1 Department of Clinical Sciences & Advanced Medicine, School of Veterinary Medicine, University of Pennsylvania, Philadelphia, PA, United States of America; 2 Department of Pathobiology, School of Veterinary Medicine, University of Pennsylvania, Philadelphia, PA, United States of America; 3 Hope Advanced Veterinary Center, Vienna, VA, United States of America; 4 Department of Clinical Studies-New Bolton Center, School of Veterinary Medicine, University of Pennsylvania, Kennett Square, PA, United States of America; 5 Department of Biomedical Sciences, School of Veterinary Medicine, University of Pennsylvania, Philadelphia, PA, United States of America; Fox Chase Cancer Center, UNITED STATES

## Abstract

Breast cancer is the most common cause of cancer-related deaths in women worldwide. Identification of reliable prognostic indicators and therapeutic targets is critical for improving patient outcome. Cancer in companion animals often strongly resembles human cancers and a comparative approach to identify prognostic markers can improve clinical care across species. Feline mammary tumors (FMT) serve as models for extremely aggressive triple negative breast cancer (TNBC) in humans, with high rates of local and distant recurrence after resection. Despite the aggressive clinical behavior of most FMT, current prognostic indicators are insufficient for accurately predicting outcome, similar to human patients. Given significant heterogeneity of mammary tumors, there has been a recent focus on identification of universal tumor-permissive stromal features that can predict biologic behavior and provide therapeutic targets to improve outcome. As in human and canine patients, collagen signatures appear to play a key role in directing mammary tumor behavior in feline patients. We find that patients bearing FMTs with denser collagen, as well as longer, thicker and straighter fibers and less identifiable tumor-stromal boundaries had poorer outcomes, independent of the clinical variables grade and surgical margins. Most importantly, including the collagen parameters increased the predictive power of the clinical model. Thus, our data suggest that similarities with respect to the stromal microenvironment between species may allow this model to predict outcome and develop novel therapeutic targets within the tumor stroma that would benefit both veterinary and human patients with aggressive mammary tumors.

## Introduction

Breast cancer is a significant cause of morbidity and mortality in both human and veterinary patients. As such, identification of reliable prognostic markers that can accurately predict patient risk will greatly reduce overtreatment of patients with less aggressive tumors and identify patients at higher risk for recurrence who would benefit from more intensive treatment. Cancer in companion animals, particularly dogs and cats, resembles cancer in humans in various ways, including: (1) its multifactorial nature, including both genetic and environmental risk factors; (2) its latency, clinical manifestation and metastatic potential; (3) its histopathologic features, including tumor cell heterogeneity and permissive microenvironment; and (4) shared prognostic markers and response, as well as resistance to therapeutics. Thus, a comparative approach to studying spontaneously occurring mammary cancer has advantages to improving clinical care across species [1].

Breast cancer is the most common cancer in both women and female dogs worldwide [2]. Although feline mammary tumors (FMT) are the 3^rd^ most common neoplasm in female cats, they account for 12% of all neoplasms and 17% of those in female cats [3]. Notably, more than 80% of FMT are malignant and extremely aggressive, with the median overall survival time of only 8–12 months following diagnosis in most studies [4–8]. Despite the fact that a relatively large number of studies have attempted to identify prognostic markers and therapeutic targets (more than 200 papers have been published on FMT) [9], FMT remains one of the most common causes of cancer-related deaths in older female cats, similar to women [10]. FMTs have been validated as a valuable preclinical translational model for the study of human breast cancer [11–13]. FMT are particularly ideal models for extremely aggressive TNBC human breast cancers, as most FMT are hormone-receptor negative [14]. The metastatic pattern (primarily to regional lymph nodes and lungs) of these aggressive FMT are the same as in humans and histologic features are more similar to human tumors than to those observed in the more low-grade canine mammary gland tumors and those in murine models [1, 11]. As in human hormone-independent breast cancers, surgery is often insufficient to cure FMT, with high rates of recurrence after complete resection [15–17]. Due to the aggressive nature of these tumors, early detection and more effective treatment options are critical for preventing metastasis, and improving survival time [17, 18].

Although FMTs are generally considered aggressive, as in humans with hormone-independent cancers, survival times can vary significantly (recently reviewed by Zappulli et al [9]). Traditionally, the Elston and Ellis (EE) grading system, adopted from human medicine and based on nuclear pleomorphism, mitotic count and tubule formation, has been used to grade FMT. While tumor grade was found to be significantly correlated with overall survival and disease-free survival in some studies [8, 19], another study suggests that grading system may not be a good prognostic indicator for grade II tumors [4] and a more recent study suggests that none of the grades in the EE grading system are correlated with survival time [20]. A modified EE (MEE) grading system, based on lymphovascular invasion, nuclear form and mitotic count appears to have superior predictive value. Unfortunately, even when grade is paired with additional clinical parameters such as age, primary tumor size and metastasis, it is clear that FMT patients need better prognostic markers to more accurately guide therapy and targeted therapies to improve survival.

Until relatively recently, investigations of cancer development and progression have focused on mechanisms within neoplastic cells that drive uncontrolled growth and metastasis. In a paradigm shift, we now realize the growth and spread of cancer also depends on the support of non-malignant cells and the extracellular matrix (ECM), particularly collagen, in the surrounding tumor stroma collectively referred to as the tumor microenvironment (TME). Overall collagen abundance correlates with both an increased breast cancer risk [21–24] and poor prognosis [25–28], and the organization and stiffness of the collagen matrix are key mediators of mammary tumor growth and invasion [29–33]. Mammary fibrillar collagen appears to activate signaling pathways that stimulate tumor cell proliferation and progression [29]. Notably, density and organization have the ability to directly influence tumorigenesis and tumor progression in both tumor-permissive and restrictive manners [33, 34] in both veterinary and human breast cancer patients, as well as murine models [25]. In addition, imaging modalities such as label-free two photon second-harmonic generation (SHG) imaging has allowed for the visualization of the specific configuration changes that occur during collagen remodeling as tumors progress. Novel imaging-processing technology has enabled the quantification of collagen fiber number and morphology, both of which have been shown to be altered in tumorigenesis and tumor progression [35–37]. Furthermore, collagen realignment and straightening has been linked to tumor invasion, metastasis and poorer prognosis in human breast cancer [38, 39].

In our recently published study [40], we identified collagen signatures in canine malignant mammary tumors that could serve as prognostic biomarkers. Specifically, lack of a defined tumor-stromal boundary and an increased collagen fiber width were associated with poor survival independent of tumor grade, patient stage, ovariohysterectomy status at the time of mammary tumor excision, and histologic evidence of lymphovascular invasion. Given that FMT are notoriously desmoplastic and biologically aggressive, we hypothesized that additional collagen fiber parameters such as length, straightness, fiber number, and SHG integrated density may also confer prognostic value in FMT. The aim of this study is to identify potential predictive biomarkers that can ultimately guide clinical care of cats with mammary carcinoma and identify tumor-permissive stromal features that may be targeted to improve clinical outcomes.

## Materials and methods

### Case selection and medical record review

Thirty female cats that underwent surgical excision for histopathologically confirmed mammary adenocarcinoma between November 11, 2002, and June 21, 2016, diagnosed through the section of Pathology at the Veterinary Hospital of the University of Pennsylvania (PennVet) and for which medical records from PennVet or referring veterinarians could be obtained, were included in this study. Biopsy samples were collected during standard of care procedures for the diagnosis and treatment of mammary carcinomas. All data in this study were obtained from materials collected in the course of routine clinical care, including residual FFPE tumor samples and medical records. Medical records from PennVet or referring veterinarians were used to obtain clinical information. Paraffin embedded tissues were obtained from PennVet for sectioning and imaging. Retrospective studies are exempt from review by the University of Pennsylvania’s Institutional Animal Care and Use Committee and the Veterinary School’s Privately Owned Animal Protocol Committee. Information collected from the medical records of cats included in the study consisted of age at the time of surgery, breed, whether an ovariohysterectomy (OHE) had been performed prior to or at the time of surgery, and the date of surgery. The number, location (right, left, bilateral and specific gland(s), if indicated) and largest diameter of the tumor was recorded. Staging (including lymph node evaluation, thoracic radiographs or abdominal ultrasonography) was recorded. Cats were excluded if there was documented previous FMT (if the biopsy represented recurrence). Although the majority of cats (24/30) had thoracic radiographs to rule out metastatic disease prior to surgical excision, thoracic radiographs were not obtained in six cats prior to surgery. Cats with distant metastases were not eligible/were not included in this study. However, data regarding regional lymph node status was collected and included in the analysis.

### Histopathologic review

Feline mammary gland carcinoma biopsies were acquired from the biopsy service archives of the PennVet Diagnostic Laboratory at the University of Pennsylvania School of Veterinary Medicine. To avoid inter-observer variability in histological interpretation that has been documented in recent veterinary studies [41, 42], all biopsies were reviewed by a single board-certified veterinary pathologist (ACD). Tumors were characterized by histologic type and were graded, based on the modified Elston-Ellis grading system which categorizes grade based upon the parameters of lymphovascular invasion, nuclear form and mitotic count [20]. In addition to type and grade, specific histologic features recorded were surgical margins (incomplete, clean-narrow (<3mm), or clean-wide (≥3 mm)), lymphovascular invasion and mitotic count (number of mitotic figures in 10 consecutive high-power fields). Lymphovascular invasion was considered to be positive, even in the absence of lymphovascular invasion on primary tumor histology, if cats had confirmed lymph node metastasis.

### Post-surgical outcome data

Information on the cats regarding the use of post-operative chemotherapy, subsequent local or distant metastasis, and cause of death was obtained through review of medical records. Disease-free survival (DFS) was established as the time interval in days from the date of the surgery to the date of any event associated with their cancer or if no cancer-associated event, then to the date of death for any reason. This included documented lymph node metastasis, new regional lymphadenopathy, documented distant metastasis or the development of new or recurrent mammary tumors. Mammary tumor recurrence was defined as regrowth of a tumor in the same region as the original resection site (local recurrence) or as a new tumor(s) when subsequent masses were noted in a region distant from the original tumor(s). Survival time (ST) was established as the time in days from the date of the surgery to the date of death for any reason. Only two cats in the study were still alive at their last known veterinary visit (these were censored for outcome analysis). Tumor-related death was defined as euthanasia or death resulting from local tumor recurrence, new tumor development, or regional or distant metastasis. Additionally, death or euthanasia not associated with new tumors or documented local or distant recurrence was classified as suspect tumor-associated death based on the clinical signs and the results of the diagnostics suggestive of metastatic disease performed at the final veterinary visit. The clinical and survival details are summarized in S1 Table for each cat.

### SHG image acquisition

SHG imaging of the fibrillar collagen was acquired using a Leica SP8 confocal/multiphoton microscope (Leica Microsystems, Inc., Mannheim, Germany), as previously described [40]. Briefly, the Coherent Chameleon Ultra II Ti:Sapphire laser (Coherent Inc., Santa Clara, CA) was tuned to 910 nm and SHG (backward) signal collected on a nondescanned hybrid detector configured to capture wavelengths at 455 nm (20x (1.0 NA) water immersion objective). Areas on the slide containing carcinoma were identified and marked by a board-certified veterinary pathologist (ACD) on serial hematoxylin and eosin (H&E) stained slides. SHG images were taken within these marked regions to ensure that intratumoral areas would be imaged as opposed to the tumor periphery, extratumoral tissue or areas of extensive necrosis. One or two non-overlapping SHG images (one z-plane) were taken at each marked location (5–7 separate areas per tumor) on a corresponding unstained slide (4–5 μm thick section) for a total of 5–12 images per tumor. Each image taken was 1024x1024 pixels (553.57 μm x 553.57 μm). Imaging parameters (laser power 22.4%, Gain: 90.8%, Offset: 51.90%) were kept identical between imaging sessions to allow for comparable image analysis quantification. The autofluorescence was subtracted from the original SHG images as previously described [40].

### Collagen parameters

We evaluated collagen density from SHG images using Fiji Image Analysis software which measured the integrated density of each SHG image, which includes intensity of each pixel and % area of positive SHG signal. For tumor-stromal boundary quantification, the SHG images were evaluated by six individuals as to whether the predominant pattern in the image was characterized by distinct tumor-stoma boundaries or a lack of discernable boundaries. Image order was randomized and evaluators were blinded with respect to tumor identity. Each evaluator had the option to score each image as either a 1, 0 or N. A score of 1 indicated that discrete tumor-stroma boundaries were the principal pattern observed. A score of 0 indicated that distinct divisions between the tumor cells and stromal collagen were not observed in the majority of the image. An “N” was reserved for images in which there was not enough collagen present to score it appropriately. Images were excluded from analysis if >50% of reviewers scored the image with an “N.” This resulted in the exclusion of 18 out of the total 286 images. The scores for each image were averaged together and the average score for each tumor was then calculated. To analyze the collagen fiber qualities (number, straightness, width and length) CT-FIRE was used, as previously described [40]. The CT-FIRE program identified all of the collagen fibers within an image, and analyzed each fiber for length, width, and % of fibers that were straight [35, 40]. The value for an image was the average of all the fibers in that image, and then all the image values for each tumor were averaged to give one value per tumor.

### Statistics

Descriptive analyses included computation of means and standard deviation for normally distributed continuous variables and tabulation of categorical variables. Shapiro-Wilk test was used to assess normality of data. Skewed data were summarized using median and interquartile range (IQR). Frequency counts and percentages were used for categorical variables such as signalment and others. Besides performing subsequent analysis with continuous data, collagen parameters were converted into categorical data, and the data sets were divided into two groups: lower and higher than the mean [40]. All descriptive analyses were performed using GraphPad Prism 5 (La Jolla, CA).

Inference statistical analysis was conducted in three steps. First, an exploratory univariate cox regression was used to assess the association between the outcomes (ST and DFS) and the independent variables. The assumption of proportional hazards was tested based on Schoenfeld residuals. Independent variables showing association with the outcome with p<0.2 were included in the subsequent analysis. Second, each combination of collagen signature variables was investigated in a well-specified base ST model that contained the significant clinical non-collagen signature variables (grade and surgical margins) in a step-wise fashion. Akaike information criterion, Bayesian information criterion, Harrell’s C concordance statistic and Somer’s D concordance statistic were evaluated to identify a best-fit model containing both clinical and collagen fiber parameters. Third and final, the best-fit models were used for reporting the inference findings. All inference analyses were conducted using either Stata 14.1MP, StataCorp, State College TX, with two-sided tests of hypotheses and a p-value < 0.05 as the criterion for statistical significance.

## Results

The cohort comprised 30 female cats with surgically excised, histologically confirmed grade I (N = 2), grade II (N = 6) or grade III (N = 22) mammary carcinomas. Clinical, histopathological, and outcome information for the cats in this study can be found in S1 Table. The mean age at diagnosis was 12.0 +/- 2.9 years. Domestic short hair was the predominant breed (80%; N = 24), with the remainder of cats listed as Maine coon (N = 1), domestic long hair (N = 2), ragdoll (N = 2) and Persian (N = 1) in medical records. OHE status of the patient and the largest diameter of the mammary mass was recorded from medical records. Regional lymph node biopsy confirmed regional metastasis at the time of surgery in 27% (8/30) of cats. Tumor type was characterized as simple carcinoma (n = 20), invasive micropapillary carcinoma (n = 1), solid carcinoma (n = 6), anaplastic carcinoma (n = 1) or intraductual papillary carcinoma (n = 2). Half (15/30) of the biopsies revealed evidence of lymphovascular invasion. Three cats had incomplete surgical margins, ten had clean-narrow surgical margins, and sixteen had clean wide surgical margins. Surgical margins could not be evaluated in one cat as only a portion of the resected tumor was submitted for evaluation. Twelve cats had local recurrence at the site of their previously excised mammary tumor, two cats had new mammary tumors and four cats had both local recurrence as well as new mammary tumor growth. Eight of the thirty cats underwent adjuvant chemotherapy (doxorubicin). For all cats, mean DFS and ST were 398 and 473 days, respectively.

**Fig 1:**
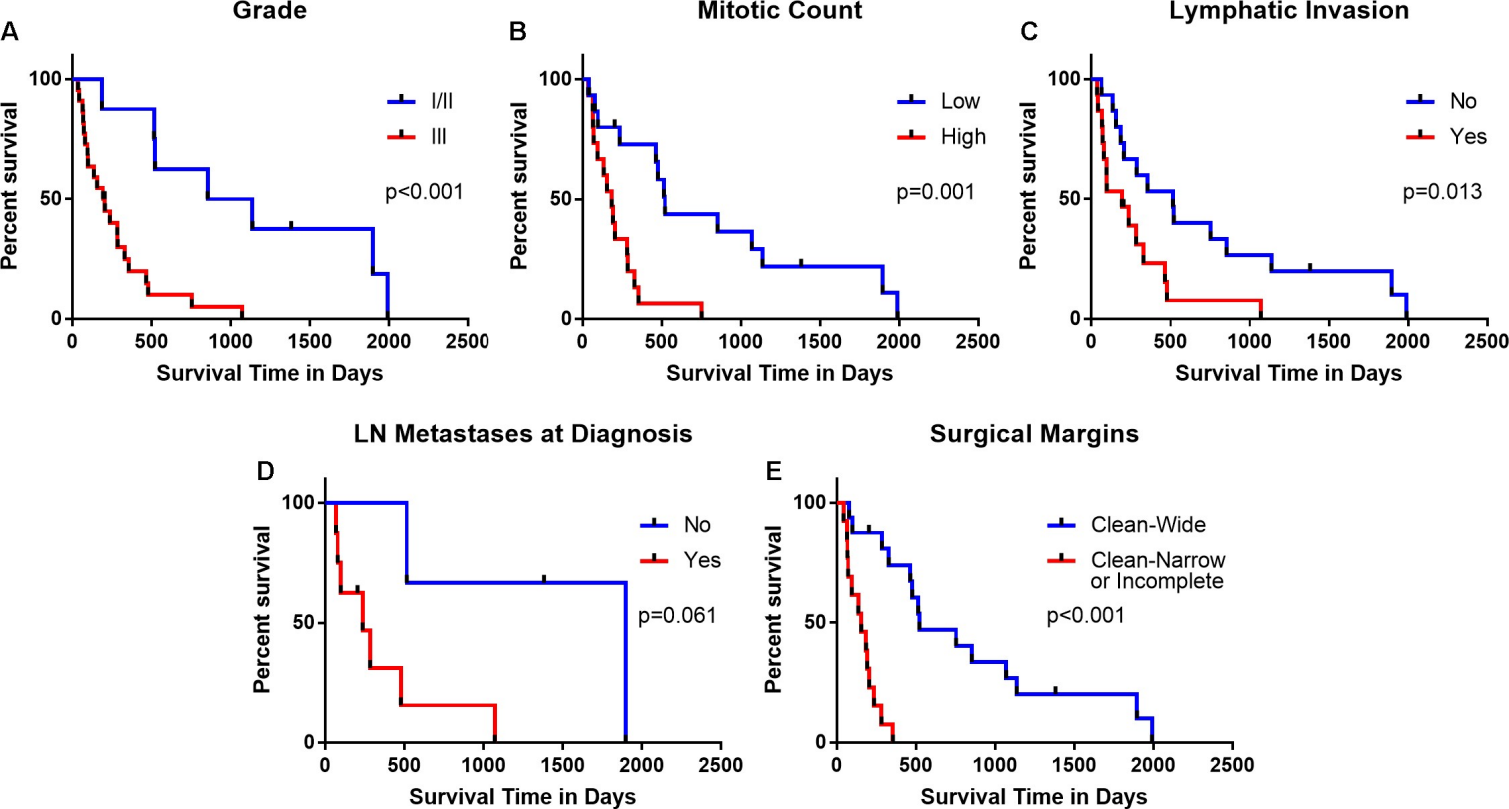
Feline mammary tumor clinical parameters predict outcome. Kaplan-Meier survival curves using cox regression univariate analysis to evaluate whether clinical parameters significantly impacted survival. (A) Grade: I/II vs. III (p<0.001, hazard ratio 2.495, 95%CI 1.493–4.168); (B) Mitotic Count: low (lower than the mean) vs. high (higher than the mean) (p = 0.001, hazard ratio 4.484, 95%CI 1.785–11.262); (C) Lymphatic Invasion: no vs. yes (p = 0.013, hazard ratio 1.658, 95%CI 1.114–2.469); (D) Lymph Node (LN) Metastases at Diagnosis: no vs. yes (p = 0.061, hazard ratio 7.825, 95%CI 0.907–67.532); (E) Surgical Margins: clean-narrow or incomplete vs. clean-wide (p<0.001, hazard ratio 0.302, 95%CI 0.170–0.539).

To investigate which clinical parameters obtained from medical records at the time of diagnosis could predict survival in our cohort of patients with FMT, we examined whether Grade category (I/II vs III), mitotic count (continuous variable), lymphatic invasion (yes vs no), tumor diameter (continuous variable based on largest diameter), lymph node metastases at diagnosis (yes vs no), or surgical margins (clean-wide vs clean-narrow or incomplete) were associated with the outcomes, specifically ST or DFS using Cox regression univariate analysis (Table 1). Grade, mitotic count, lymphatic invasion, and surgical margins were significant predictors of ST, as well as DFS. In addition, lymph node metastases at diagnosis also significantly predicted DFS, but did not predict overall ST. Grade (incorporating lymphatic invasion and mitoses), mitotic count, lymphatic invasion, lymph node metastases at diagnosis, and surgical margins are parameters often used by clinicians to predict patient survival. To better illustrate how these factors impacted survival, the Kaplan-Meier product limit method and the log rank test were used to compare outcome across variables (Fig 1). Comparing Grade I/II to III provided significant differences in ST (median ST = Grade I/II: 995.5 days; Grade III: 198.5 days), however, it is clear that prognostication using grade alone fails to predict clinical outcome in all cats in each group (Fig 1A). As an example, most cats with Grade III tumors died within a year after diagnosis, but nearly one in five cats with grade III tumors survived longer than twice the median survival of all cats with grade III tumors post-surgery. Similarly, mitotic count, a component of Grade, significantly affected survival where higher mitotic counts were associated with shorter survival (Fig 1B). Survival times for cats with or without lymphatic invasion exhibited substantial overlap, although there was a significantly shorter ST for those cats with confirmed lymphatic invasion (Fig 1C). Despite this, it should be noted that three cats with lymphatic invasion (20%) survived well beyond a year after surgery (mean ST for these cats was 22 months). Interestingly, documented lymph node metastasis at the time of surgery did not have a significant impact on ST in this study (Fig 1D). The absence of clean-wide surgical margins was a strong clinical predictor of decreased survival time (Fig 1E), as all cats with clean-narrow or incomplete margins died before the median survival time of cats with clean-wide margins (median ST = clean-wide: 522 days; clean-narrow or incomplete: 155 days). However, 35% of cats with clean-wide margins also died within a year after surgery. Finally, we evaluated OHE status, tumor type and surgery type, but these parameters did not reach, or trend toward, significance in our cohort of 30 cats.

**Table 1:** Univariate analysis of clinical parameters.

	Parameter	Comparison	Hazard Ratio	Standard Error	p value	95% Confidence Interval	
**ST**	Grade	I/II vs. III	2.495	0.653	<0.001	1.493	4.168
	Mitotic Count	Continuous Values	1.025	0.006	<0.001	1.012	1.037
	Lymphatic Invasion	No or Yes	1.658	0.337	0.013	1.114	2.469
	Tumor Diameter	Continuous Values	0.790	0.208	0.372	0.472	1.324
	LN Mets at Diagnosis	No or Yes	7.825	8.605	0.061	0.907	67.532
	Surgical Margins	Clean-Narrow or Incomplete vs. Clean-Wide	0.302	0.089	<0.001	0.170	0.539
**DFS**	Grade	I/II vs. III	2.521	0.634	<0.001	1.539	4.127
	Mitotic Count	Continuous Values	1.021	0.007	0.002	1.008	1.035
	Lymphatic Invasion	No or Yes	1.792	0.352	0.003	1.219	2.634
	Tumor Diameter	Continuous Values	0.801	0.202	0.378	0.489	1.312
	LN Mets at Diagnosis	No or Yes	8.494	9.247	0.049	1.006	71.740
	Surgical Margins	Clean-Narrow or Incomplete vs. Clean-Wide	0.267	0.090	<0.001	0.137	0.517

ST, survival time; DFS, disease-free survival; LN, lymph node

**Fig 2:**
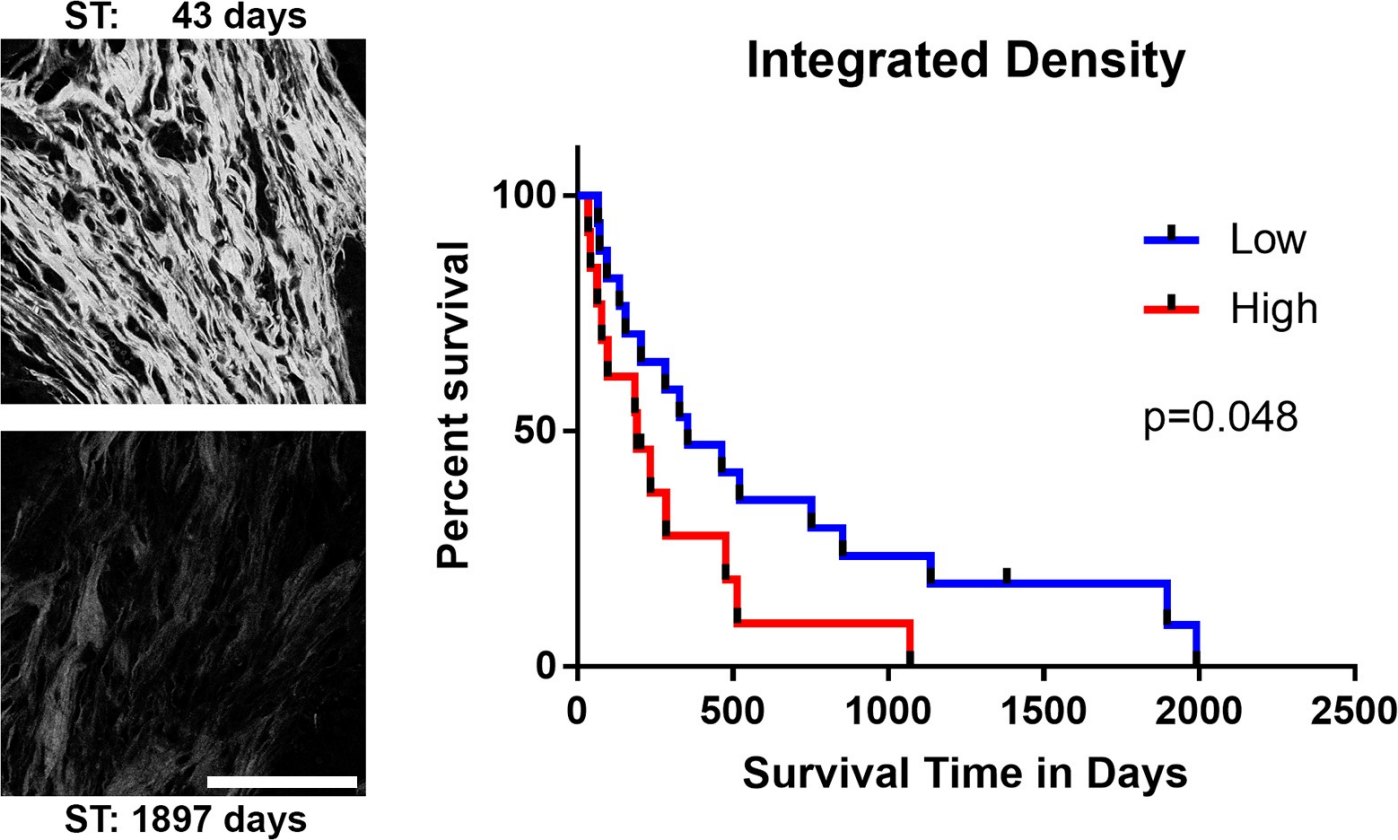
Fibrillar collagen density in feline mammary tumors. Representative images of SHG signal (white) in a tumor from a cat with short survival time (top) and a cat with a long survival time (bottom) following mammary tumor excision. Kaplan-Meier survival curve for 30 cats with collagen integrated density higher or lower than the mean integrated density value. Univariate cox regression was used to evaluate whether the collagen density significantly impacted survival (p = 0.048, hazard ratio 2.125, 95%CI 1.007–4.483). All images in figure are at the same scale. Scale bar: 100μm.

**Fig 3:**
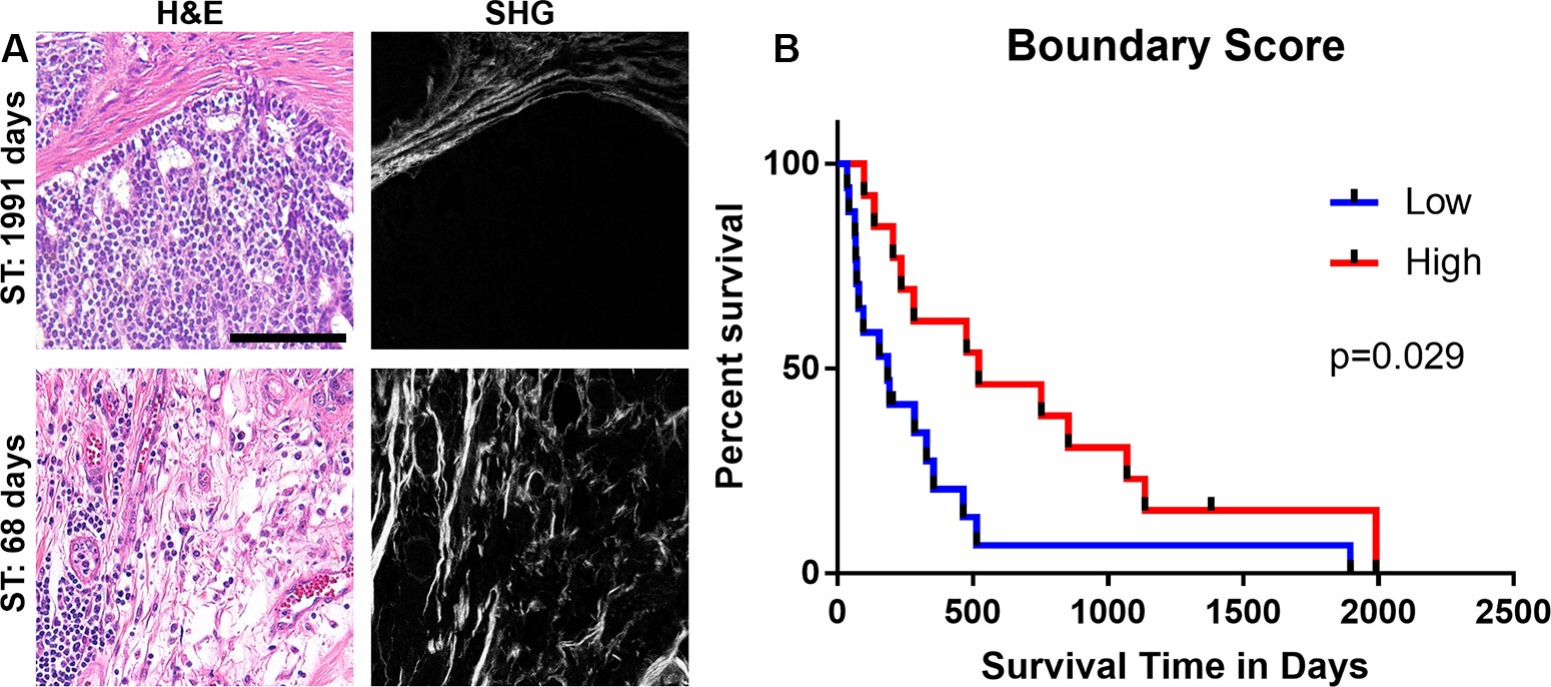
Absence of a tumor-stromal boundary predicts poor outcome in feline mammary tumors. (A) H&E and corresponding SHG (collagen, white; from serial sections) in representative images of tumors obtained from a long-lived patient (ST = 1991 days) with a high boundary score (A; top) and from a short-lived patient (ST = 68 days) with a low boundary score (A; bottom). Six observers graded each image as either (1) discrete tumor-stroma boundaries were the principal pattern observed or (0) tumor-stromal boundaries were not the principal pattern. The boundary score is the average evaluator score for each image, averaged for each tumor. (B) Kaplan-Meier survival curve for 30 cats with boundary scores higher or lower than the mean boundary score. Univariate cox regression was used to evaluate whether the boundary score significantly impacted survival (p = 0.029, hazard ratio 0.385, 95%CI 0.164–0.906). All images in figure are at the same scale. Scale bar: 100μm.

**Fig 4:**
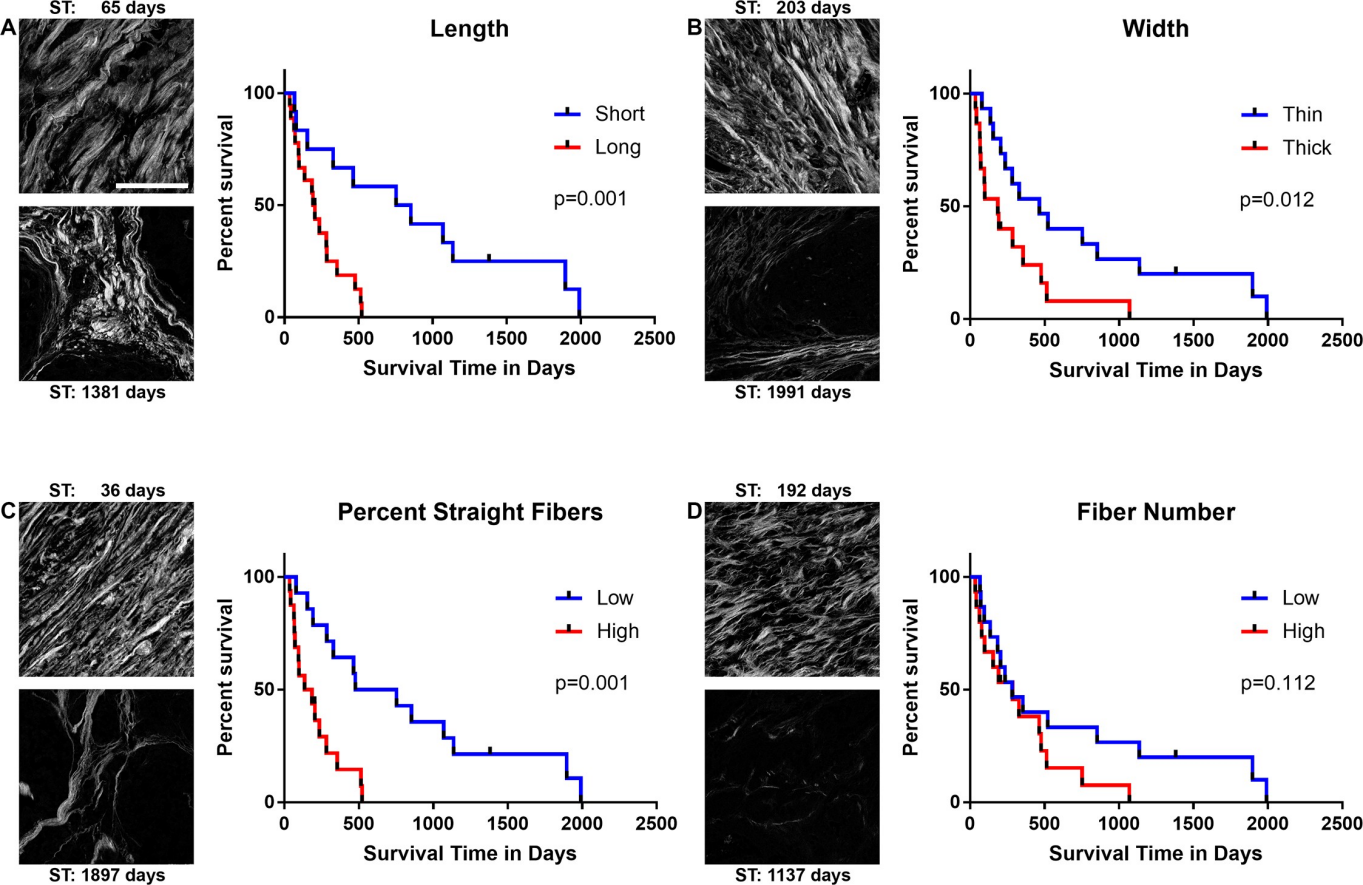
Collagen fiber characteristics in feline mammary carcinoma biopsies predict survival. Collagen fiber characteristics were quantified using data analysis software (CT-FIRE). Data were compared to ST using Kaplan-Meier survival curves, comparing lower than the mean and higher than the mean groups for collagen fiber (A) length, (B) width, (C) straightness, and (D) number. Univariate cox regression was used to evaluate whether the collagen fiber parameters significantly impacted survival (length: p = 0.001, hazard ratio 4.597, 95%CI 1.849–11.428; width: p = 0.012, hazard ratio 2.650, 95%CI 1.242–5.654; straightness: p = 0.001, hazard ratio 4.390, 95%CI 1.890–10.197; number: p = 0.112, hazard ratio 1.827, 95%CI 0.869–3.838). For each parameter, example SHG images with survival times are shown from the low and high groups. All images in figure are at the same scale. Scale bar: 100μm.

To examine whether fibrillar collagen characteristics in FMT biopsy samples could predict survival outcome, SHG imaging was performed on histologic biopsy samples from 30 cats with mammary carcinoma (Figs 2–4). Given that increased collagen density has been shown to correlate with tumor invasiveness and poor clinical outcome in human breast cancer [21–25] and canine mammary tumor [40] tissues, we examined the overall fibrillar collagen levels in feline mammary tumors by quantitating the integrated density of the SHG signal to determine whether higher collagen density was associated with poor survival. A Kaplan-Meier survival curve and cox regression analysis showed a significant difference in ST between cats with tumors that had higher than the mean collagen density (median ST = 192 days) compared to those cats with tumors having a collagen density lower than the mean density (median ST = 355 days, p = 0.048; Fig 2). This difference was also significant for DFS (Table 2). While the difference in survival was significant between the two groups, half of the cats in the lower collagen density group survived for less than a year after surgery. Given that fibrillar collagen may play both tumor-permissive and tumor-restrictive roles [43], we next sought to determine if collagen fiber characteristics could better predict clinical outcomes in cats with FMT.

**Table 2:** Univariate analysis of collagen parameters.

	Parameter	Comparison	Hazard Ratio	Standard Error	p value	95% Confidence Interval	
**ST**	Collagen Integrated Density	Lower or Higher than Mean	2.125	0.809	0.048	1.007	4.483
	Boundary Score	Lower or Higher than Mean	0.385	0.168	0.029	0.164	0.906
	Collagen Fiber Length	Lower or Higher than Mean	4.597	2.136	0.001	1.849	11.428
	Collagen Fiber Width	Lower or Higher than Mean	2.650	1.025	0.012	1.242	5.654
	Collagen Fiber Number	Lower or Higher than Mean	1.827	0.692	0.112	0.869	3.838
	Collagen Fiber Straightness	Lower or Higher than Mean	4.390	1.888	0.001	1.890	10.197
	Collagen Integrated Density	Continuous Values	>1.000	0.000	0.006	<1.000	>1.000
	Boundary Score	Continuous Values	0.199	0.126	0.011	0.057	0.691
	Collagen Fiber Length	Continuous Values	1.085	0.026	0.001	1.035	1.136
	Collagen Fiber Width	Continuous Values	3.338	1.266	0.001	1.587	7.021
	Collagen Fiber Number	Continuous Values	1.001	0.000	0.032	1.000	1.002
	Collagen Fiber Straightness	Continuous Values	1.420E+13	8.610E+13	<0.001	1.010E+08	2.000E+18
**DFS**	Collagen Integrated Density	Lower or Higher than Mean	2.756	1.106	0.012	1.255	6.051
	Boundary Score	Lower or Higher than Mean	0.422	0.170	0.032	0.192	0.930
	Collagen Fiber Length	Lower or Higher than Mean	3.715	1.715	0.004	1.503	9.182
	Collagen Fiber Width	Lower or Higher than Mean	4.004	1.750	0.002	1.700	9.431
	Collagen Fiber Number	Lower or Higher than Mean	1.918	0.727	0.086	0.912	4.032
	Collagen Fiber Straightness	Lower or Higher than Mean	3.957	1.792	0.002	1.629	9.612
	Collagen Integrated Density	Continuous Values	>1.000	2.710E-08	0.001	<1.000	>1.000
	Boundary Score	Continuous Values	0.236	0.145	0.019	0.071	0.785
	Collagen Fiber Length	Continuous Values	1.115	0.028	<0.001	1.062	1.171
	Collagen Fiber Width	Continuous Values	4.337	1.711	<0.001	2.002	9.395
	Collagen Fiber Number	Continuous Values	1.001	0.000	0.012	1.000	1.002
	Collagen Fiber Straightness	Continuous Values	2.380E+13	1.610E+14	<0.001	4.090E+07	1.390E+19
	Collagen Integrated Density	Lower or Higher than Mean	2.756	1.106	0.012	1.255	6.051

ST, survival time; DFS, disease-free survival.

In our previous study examining the regulatory role of collagen on mammary tumor behavior in dogs, a new prognostic variable, boundary score, was developed, which we showed positively correlated with survival in canine malignant mammary tumors independently of clinical parameters [40], thus we included this parameter in our feline analysis. Comparing H&E images with corresponding regions of SHG images clearly shows the dramatic difference between the presence of a defined tumor-stromal boundary in biopsy samples from cats with longer and shorter survival times (Fig 3A). Quantitative analysis of these scores showed a significant difference in survival between cats with tumors with a boundary score higher than the mean and tumors with low boundary scores, (ST: p = 0.029; Fig 3B; DFS: p = 0.032; Table 2). As with dogs, FMT progress from less aggressive tumors with clear boundaries between tumor cells and stroma, to more aggressive tumors where the boundaries have been lost and there is intermingling of stroma and tumor cells throughout the tumor.

As in our canine study [40], collagen fibers were also characterized in FMT biopsies on the basis of length, width and straightness using data analysis software (CT-FIRE; Fig 4A–4C). We found that fiber length, width, and straightness were all significantly associated with survival, as cats with tumors with long, thick, straight fibers had the poorest survival (both ST and DFS, Table 2). For this study, we also evaluated average fiber number per image was evaluated with respect to survival (Fig 4D). Fiber length and straightness (Fig 4A and 4C) were particularly accurate predictors of survival, as all of the cats (1 cat censored) with longer or straighter than the mean fibers did not survive beyond the median survival of cats with tumors containing collagen fibers that were shorter or wavier than the mean (804 days for length, and 615.5 for straightness). These findings are consistent with our data examining collagen fiber characteristics and their relationship with clinical outcomes in dogs with malignant mammary tumors, which supports investigation of this role in humans and other species, and further studies into whether targeting the formation of these collagen signatures would improve clinical outcomes.

To test if the collagen parameters were independently driving the differences in outcome, multivariable analysis was used. First, a base mode was identified by testing all clinical parameters in a step-wise fashion. After all combinations were considered, grade and surgical margins were included in the final model. Next, the collagen parameters (as both continuous and categorical variables) were individually tested in a cox multivariable regression model controlling for the base model of clinical parameters (Table 3). Collagen integrated density (p = 0.040, hazard ratio >1.000), boundary score (p = 0.001, hazard ratio 0.191; categorical p = 0.022, hazard ratio 0.416), and fiber number (categorical p = 0.022, hazard ratio 3.280) were significantly associated with ST independently of grade and surgical margins. For DFS, collagen width (p = 0.003, hazard ratio 6.131; categorical p = 0.003, hazard ratio 4.473), fiber number (p = 0.008, hazard ratio 1.002; categorical p = 0.002, hazard ratio 5.102), straightness (p = 0.046, hazard ratio 3.035), integrated density (p<0.001, hazard ratio >1.000), and boundary score (p = 0.005, hazard ratio 0.169; categorical p = 0.012, hazard ratio 0.368) significantly affected outcome in a model including grade and surgical margins. Thus, nearly all of the collagen parameters we evaluated affected clinical outcome independently of the clinical parameters.

**Table 3:** Multivariable analysis.

	ST					DFS				
Parameter	Hazard Ratio	Standard Error	p value	95% Confidence Interval		Hazard Ratio	Standard Error	p value	95% Confidence Interval	
Grade	4.243	2.650	0.021	1.247	14.430	4.810	2.874	0.009	1.491	15.517
Surgical Margins	0.145	0.089	0.002	0.044	0.484	0.100	0.068	0.001	0.026	0.379
Grade	4.160	2.337	0.011	1.383	12.511	5.139	2.269	<0.001	2.163	12.210
Surgical Margins	0.153	0.102	0.005	0.042	0.562	0.135	0.098	0.006	0.033	0.557
Length	1.010	0.028	0.725	0.956	1.066	1.061	0.033	0.053	0.999	1.128
Grade	2.924	1.559	0.044	1.028	8.316	3.852	1.516	0.001	1.780	8.333
Surgical Margins	0.138	0.084	0.001	0.042	0.454	0.073	0.057	0.001	0.016	0.335
Width	2.533	1.219	0.053	0.986	6.505	6.131	3.758	0.003	1.844	20.383
Grade	2.164	1.246	0.180	0.701	6.686	2.392	0.939	0.026	1.108	5.163
Surgical Margins	0.080	0.060	0.001	0.019	0.344	0.040	0.037	<0.001	0.007	0.241
Number	1.001	0.001	0.059	1.000	1.003	1.002	0.001	0.008	1.001	1.004
Grade	5.191	2.824	0.002	1.788	15.076	8.012	4.056	<0.001	2.971	21.609
Surgical Margins	0.339	0.241	0.128	0.084	1.366	0.284	0.227	0.115	0.060	1.359
Straightness	5.560E+07	5.500E+08	0.071	0.211	1.460E+16	1.200E+12	1.670E+13	0.046	1.66	8.620E+23
Grade	3.014	1.609	0.039	1.059	8.580	3.930	1.538	<0.001	1.825	8.462
Surgical Margins	0.125	0.076	0.001	0.038	0.413	0.078	0.060	0.001	0.017	0.352
Integrated Density	>1.000	3.090E-08	0.04	<1.000	>1.000	>1.000	2.65E-08	<0.001	<1.000	>1.000
Grade	5.167	2.895	0.003	1.723	15.496	5.075	2.454	0.001	1.967	13.093
Surgical Margins	0.162	0.102	0.004	0.047	0.560	0.060	0.050	0.001	0.012	0.305
Boundary Score	0.191	0.099	0.001	0.069	0.530	0.169	0.106	0.005	0.049	0.581
Grade	4.203	2.026	0.003	1.634	10.811	4.949	2.216	<0.001	2.058	11.902
Surgical Margins	0.166	0.100	0.003	0.051	0.541	0.133	0.092	0.004	0.034	0.516
Length, ctg	2.936	1.654	0.056	0.973	8.859	1.921	0.850	0.140	0.808	4.572
Grade	3.503	1.901	0.021	1.209	10.148	4.934	1.943	<0.001	2.280	10.675
Surgical Margins	0.146	0.091	0.002	0.043	0.496	0.058	0.049	0.001	0.011	0.301
Width, ctg	1.526	0.655	0.325	0.658	3.539	4.473	2.222	0.003	1.690	11.841
Grade	5.240	2.928	0.003	1.753	15.666	6.290	3.339	0.001	2.222	17.803
Surgical Margins	0.306	0.220	0.099	0.075	1.248	0.219	0.176	0.059	0.045	1.060
Straightness, ctg	2.717	1.611	0.092	0.850	8.688	3.035	2.234	0.131	0.717	12.841
Grade	3.246	1.599	0.017	1.236	8.522	4.558	1.825	<0.001	2.079	9.990
Surgical Margins	0.115	0.067	<0.001	0.037	0.357	0.098	0.070	0.001	0.024	0.396
Integrated Density, ctg	1.893	0.773	0.118	0.850	4.214	2.228	0.982	0.069	0.940	5.284
Grade	1.649	1.024	0.421	0.488	5.571	1.582	0.715	0.310	0.653	3.837
Surgical Margins	0.055	0.044	<0.001	0.011	0.266	0.025	0.023	<0.001	0.004	0.146
Number, ctg	3.280	1.702	0.022	1.186	9.069	5.105	2.730	0.002	1.790	14.559
Grade	4.942	2.636	0.003	1.737	14.059	5.249	2.423	<0.001	2.124	12.970
Surgical Margins	0.165	0.099	0.003	0.051	0.535	0.073	0.057	0.001	0.016	0.339
Boundary Score, ctg	0.416	0.159	0.022	0.196	0.881	0.368	0.147	0.012	0.168	0.804

ctg, categorical (higher or lower than mean); ST, survival time; DFS, disease-free survival

Further, we aimed to discover if the collagen parameters could help predict outcome better than the clinical model alone, and therefore uncover the best-fit model including clinical and collagen parameters. For this, Akaike information criterion (AIC; smaller values indicate better-fit model), Bayesian information criterion (BIC; smaller values indicate better-fit model), Harrell’s C concordance statistic and Somer’s D concordance statistic (larger values indicate better-fit model) were evaluated to identify a best-fit model containing both clinical and collagen fiber parameters (Table 4). We tested the model containing the clinical parameters first, then added in the collagen parameters and tested each subsequent model. After several iterations testing all combinations, the best model for ST included grade, surgical margins, boundary score, collagen fiber length (categorical) and collagen fiber straightness (categorical). For DFS, the best-fit model included grade, surgical margins, collagen fiber width (categorical) and boundary score. These models, with collagen parameters evaluated in addition to clinical parameters, surpassed the clinical models alone.

**Table 4:** Multivariable models.

	Parameter	Hazard Ratio	Standard Error	p value	95% Confidence Interval		AIC	BIC	Harrell's C	Somer’s D
**ST**	Grade	4.243	2.650	0.021	1.247	14.430				
	Surgical Margins	0.145	0.089	0.002	0.044	0.484				
							113.492	116.226	0.777	0.553
	Grade	5.167	2.895	0.003	1.723	15.496				
	Surgical Margins	0.162	0.102	0.004	0.047	0.560				
	Boundary Score	0.191	0.099	0.001	0.069	0.530				
							109.407	113.509	0.828	0.656
	Grade	5.894	3.183	0.001	2.045	16.988				
	Surgical Margins	0.168	0.105	0.004	0.049	0.573				
	Boundary Score	0.093	0.064	0.001	0.024	0.357				
	Length, ctg	4.768	2.637	0.005	1.613	14.096				
							103.091	108.560	0.840	0.680
	Grade	11.219	8.784	0.002	2.419	52.045				
	Surgical Margins	0.317	0.200	0.068	0.092	1.090				
	Boundary Score	0.047	0.035	<0.001	0.011	0.206				
	Length, ctg	5.183	3.110	0.006	1.599	16.801				
	Straightness, ctg	3.302	1.753	0.024	1.167	9.345				
							101.027	107.863	0.862	0.724
**DFS**	Grade	4.810	2.874	0.009	1.491	15.517				
	Surgical Margins	0.100	0.068	0.001	0.026	0.379				
							117.611	120.346	0.784	0.568
	Grade	4.934	1.943	<0.001	2.280	10.675				
	Surgical Margins	0.058	0.049	0.001	0.011	0.301				
	Width, ctg	4.473	2.222	0.003	1.690	11.841				
							109.968	114.070	0.835	0.669
	Grade	5.997	2.632	<0.001	2.537	14.175				
	Surgical Margins	0.047	0.045	0.001	0.007	0.310				
	Width, ctg	3.406	1.647	0.011	1.320	8.788				
	Boundary Score	0.220	0.147	0.023	0.059	0.813				
							106.958	112.427	0.861	0.721

ctg, categorical (higher or lower than mean); AIC, Akaike information criterion; BIC, Bayesian information criterion; ST, survival time; DFS, disease-free survival. To determine the best-fit model, AIC and BIC (smaller values = better-fit) and Harrell’s C and Somer’s D (larger values = better-fit) values were analyzed.

## Discussion

Companion animals serve as useful models of corresponding diseases in humans and also improve understanding of these diseases in veterinary patients. After previously defining collagen characteristics that could predict outcome in canine mammary tumor patients, we hypothesized that collagen plays a critical role in directing the biologic behavior of FMT and certain collagen signatures could predict clinical outcomes in these feline patients. Additionally, as mammary tumors in cats are nearly always malignant and typically more aggressive than in dogs, this may in part explain the difference in the distribution across grades between cats and dogs. Notably, only 2 cats in our study had grade I tumors and the majority had grade III tumors, this is the opposite of what is seen in dogs where grade I, hormone receptor positive carcinomas are the most common and grade III tumors represent 20–30% of the cases [44, 45]. Thus, feline mammary carcinomas are superior models for aggressive forms of human breast cancer, and provide a unique opportunity to explore the role of collagen in the tumor microenvironment. Here, we present evidence that in the highly aggressive FMT, all of the collagen parameters that we evaluated affected both ST and DFS. Additionally, when evaluated in multivariable analysis including the clinical variables tumor grade and surgical margins, increased collagen fiber straightness, number, width, and density, and a lack of tumor-stromal boundary, were associated with poor outcomes. Finally, models including the clinical parameters grade and surgical margins together with collagen parameters were stronger predictors of outcome than these clinical parameters alone. While our data is compelling and provides a premise that collagens play a critical role in regulating biologic behavior of FMT, larger studies will need to be performed to confirm our results prior to incorporating our findings into clinical practice. Furthermore, larger studies will permit stratification of data by clinical parameters such as tumor type, surgical approach and the use of neoadjuvant chemotherapies to determine if collagen signature analysis may be more beneficial for certain patients, as well as to test whether these signatures can predict susceptibility to certain therapeutic interventions.

Predicting the course of disease for patients diagnosed with FMT is crucial for planning care and treatment for the patient. Currently, veterinarians and pathologists take tumor size, grade and associated mitotic count, lymphovascular invasion, surgical margins, as well as available information on lymph node involvement and distant metastasis into account when predicting outcome and recommending treatment. In our study, tumor size did not predict outcome. Given that previous studies had identified that tumor size is only predictive of outcome when the tumor is greater than 3cm [20, 46] and only two of the cats in this study had tumors greater than 3cm, this was not surprising. Although grade is often heavily relied upon as a prognostic indicator, the current grading system for FMT has been revealed to have limitations. The human Elston and Ellis system initially applied to FMT successfully predicts outcome for the majority of cats with grade I and III, but categorizes most FMT in grade II, which has the most limited predictive value [4]. Taking other factors into account, such as lymphovascular invasion and nuclear form, Mills et al. in 2015 showed that the grading system can be improved [20], and it has been confirmed that the modified Elston and Ellis system improves prognostic value in a large (342 cats with invasive mammary carcinoma) study [47]. However, in the Mills 2015 novel grading system, not many tumors are considered grade I. In our study, less than 7% of the tumors (2 cats) were grade I using this method, and both of those cats were long-lived (mean survival 996 days/~33 months). The vast majority (73%) of the tumors in our study were grade III, but 18% of these cats survived for over a year after surgery, and half of these survived more than three-times the median ST for this group. The grade II tumors (mean survival 1082 days) had highly variable survival times with half being the longest living cats in our cohort (mean survival 1756 days), but the other half dying within 1.5 years after surgery (mean survival 407 days). Identification of novel markers which could better predict outcomes for feline patients with tumors of all grades, but particularly for those patients with grade II tumors, would fill a critical clinical need.

While the human breast cancer field is identifying new prognostic markers for human patients at a fast pace, the discovery of useful biomarkers in FMT has lagged behind, although several candidates have been considered. Recently the receptor CXCR4 and its ligand CXCL12 have been studied in FMT, and decreased CXCL12 was associated with poor prognosis, but only in tumors highly expressing HER2 [48]. Staining primary tumors for Bcl-2, an anti-apoptotic protein, revealed that increased Bcl-2 positivity was associated with longer survival, disease-free survival and cancer-specific survival [49]; however only a small proportion of FMT were positive for Bcl-2 in this study, and thus this marker may only be used to prognosticate a subpopulation of feline patients. Consistent with the ability of SDF-1 to predict outcome in human patients, SDF-1 was also found to be higher in cats with FMT compared to healthy individuals, although it was unable to predict outcome [50]. Finally, sphingosine kinase-1 (SPHK1) was found to be increased in FMT vs normal mammary tissue, and levels within primary tumors correlated with grade, lymphovascular invasion, and ER negativity. However, while SPHK1 was shown to predict poor prognosis in human, this association was not confirmed in FMT [51]. While future studies may identify additional prognostic biomarkers for FMT, this study suggests not only the importance of specific collagen signatures in the pathogenesis of FMT but also their potential place in a prognostic panel to better serve patients with FMT as well as to identify targets to improve clinical care of these and other species with malignant mammary tumors in a comparative oncology approach.

Although human breast cancer research has looked at breast density, determined in part by collagen density, as a marker of poor outcome for patients for decades [23], it was not until more recently that a causal link between collagen density and tumor progression was identified [25, 39]. In our study, like in women and dogs with mammary gland tumors, collagen density predicts survival of cats with mammary carcinoma. In addition to collagen density, its organization and alignment has been shown to be important regulators of cancer progression [35, 36, 38, 39, 52]. Furthermore, individual collagen fiber characteristics may modulate cell activity and fate in the tumor microenvironment. For example, an increased collagen fiber length was found in pancreatic ductal adenocarcinoma compared to non-neoplastic tissue [53]. Increased fiber length has been correlated with poor patient survival in several types of other cancers, including head and neck, esophageal and colorectal [54]. In gastric cancer, collagen density as well as fiber width, length, straightness and alignment were all found to be increased compared to non-neoplastic tissues [55]. Of these, width of collagen fibers was the most powerful parameter in predicting 5-year overall survival (with increased fiber width associated with poor outcome). Collagen fiber straightness seems to be important in mammary tumors, as collagen fibers were straightest in the intratumoral region and curliest farther from the tumor cells in the extratumoral region in human breast tumor samples [56].

Our canine study was the first to link tumor-stromal (collagen) boundary score to clinical outcome [40]. This parameter was established after trying to identify tumor-stromal boundaries in order to quantify tumor-associated collagen signatures (TACS) [25, 33, 38, 39]. We suggested that the absence of a clear tumor-stromal boundary could be the progression past TACS-3, which was then considered the invasive and aggressive tumor signature. Similar to canine mammary tumors, a well-defined tumor-stromal boundary is strongly associated with improved FMT outcomes, independent of clinical parameters grade and surgical margins. This suggests that the incorporation of boundary scores, determined by SHG imaging of routine biopsy sections, into a panel of known predictive markers could improve prognostication. While we used a system of evaluators to score boundary, prior to incorporation of SHG analysis into clinical prognostic panels, determination of boundary scoring will likely need to become automated such that an unbiased and time-efficient analysis of this survival predictor can be validated. Given its predictive capacity in both heterogeneous canine and typically more biologically aggressive feline malignant mammary tumors, our data suggest that boundary score could improve prognostication in other species as well, including humans.

While our study confirms that collagen fiber length, straightness, number, width, density, and lack of a tumor-stromal boundary are all associated with poor outcomes in FMT patients, it is probable that additional prognostic information could be acquired from collagen based on recently described imaging techniques and analytics. Collagen quantity, uniformity and organization measured with SHG and ImageJ software in both the forward and backwards directions can identify human mammary tumor subgroups [57] and collagen quantity and uniformity from SHG images can predict outcome in luminal breast cancer [58]. Similarly in human vulvar squamous cell carcinoma, increased collagen quantity and uniformity correlated with the presence of distant metastasis, suggesting that these collagen parameters could be used for prognosis in this type of cancer as well [59]. In murine colorectal cancer, SHG paired with metabolic *in vivo* imaging could predict response to chemotherapy [60], which could tailor personalized oncologic therapies. Recently, new collagen SHG parameters, chirality, 3D orientation, and a polarization-sensitive SHG microscopy technique, polarization-in, polarization-out (PIPO) SHG, have been leveraged to differentiate normal and neoplastic pancreatic tissues [61, 62].

Although this initial study supports further investigation and validation of the use of SHG imaging to improve prognostication in FMT patients, it should be acknowledged that this imaging modality is not readily available to all Veterinary pathologists and oncologists. Assessment of collagen fiber features has also been performed using picrosirius red imaging with polarization, a technique with wider accessibility. The two staining techniques have been directly compared [63], and while both were shown to provide similar collagen information, fiber length and width did not significantly correlate between the two imaging techniques. In light of our findings of the importance of fiber width and length in predicting clinical outcome in our cohort of cats, it is unlikely PSR polarization microscopy may provide a suitable alternative for imaging predictive collagen signatures in FMT, although that hypothesis has yet to be tested. While SHG imaging is not yet readily accessible to many pathology laboratories as a component for diagnostic testing, it is not uncommon for specialized diagnostics to be performed by outside institutions in Veterinary Medicine, which will become advantageous if larger studies confirm our findings. Given that SHG analysis can be performed on unstained FFPE slides, this would allow samples to be obtained from available archived samples (after the initial diagnosis is made by the consulting veterinary pathologist) and without special shipping requirements, facilitating both translational research studies and eventually clinical diagnostics at remote sites.

From our findings, we conclude that the density and organization of the collagen in FMT directs tumor aggressiveness and therefore clinical outcomes. Certainly, several mechanisms by which collagen density and stiffness promote metastasis and shorten survival and the mechanisms contributing to their formation and cellular responses have been suggested, including changes in collagen type proportions [64, 65] and via alterations in integrin signaling [66, 67]. Increased density and linearization of collagen at the forefront of breast cancer cell invasion into surrounding normal tissue correlates with higher stromal stiffness and cellular mechanosignaling [68]. Additionally, tumor cells have been shown to migrate and invade tissues more efficiently along aligned collagen fibers [38, 69]. Mouse tumors in a dense collagen environment had increased lung metastases and the tumor collagen fibers were perpendicular to the bulk of the tumor, projecting into the surrounding fat [70], further connecting tumor organization with outcome and tumor progression. However, the direct effects of changes in collagen fibers like length, straightness, and width on tumor and stromal cell behaviors are just beginning to be understood [71]. The thickening and straightening of fibers could lead to changes in ECM porosity, effectively limiting cells ability to randomly move through the tumor. This could enhance the cells ability and/or requirement to leave the tumor and invade adjacent tissues. Undoubtedly, these changes also impact the biomechanical properties of the tumor microenvironment. In general, changes in the ECM organization will also change the cells within the tissue including their interactions with the ECM and other cells [66]. Recently, methods to directly test how collagen and its organization affect cell properties have been developed, including electrospun fibers made to mimic the tumor microenvironment, which promoted normal epithelial cells to adapt a mesenchymal morphology [72] and *in vitro* gels that mimic tumor-associated collagen architectures with large, aligned collagen bundles, which elicited cancer cell contact guidance and enhanced their directional migration [73]. While the collagen organization in FMT likely affects the neoplastic and cancer associated fibroblasts within the tumor, immune cells may also alter their behavior based on tumor ECM and collagen organization. Matrix stiffness may provide a barrier that prevents infiltration of immune cells into the tumor and also promotes a tumor-suppressive microenvironment for intratumoral cells [74–77]. Activation of the mechanosensitive transcriptional regulator YAP (yes-associated protein) in pancreatic, prostatic, hepatic and ovarian cancer cells has been shown to be essential for efficient cytokine-mediated myeloid derived suppressor cell (MDSC) recruitment and infiltration. Similarly, YAP activity in human lung cancer cells that disrupts T cell function may elicit mechano-mediated immune evasion [78, 79]. Substrate stiffness also may directly influence the polarization state, function and mode of migration of macrophages [80]. Future studies will evaluate the influence of collagen biophysical, biomechanical and biochemical influences on immune infiltration and activities in FMT. These questions are central to understanding mechanisms by which collagen affects not only biologic behavior of tumors that impacts survival, but will inform the development of novel therapies for breast cancer.

We propose that several features of collagen in the tumor microenvironment, specifically collagen density, organization, and fiber characteristics affect biologic behavior of mammary tumors and can predict outcome in FMT. In this relatively small study, the most predictive multivariable model for overall survival included the clinical parameters grade and surgical margins, as well as the collagen parameters boundary score, collagen fiber length and collagen fiber straightness. Thus, validation of these findings in a larger study will help to improve accurate prediction of the clinical course for cats with mammary cancer. Importantly, this may improve delivery of clinical care in individual patients by determining which patients are most at risk for recurrence of disease. Finally, a better understanding of the mechanisms by which these tumor-permissive and restrictive collagen signatures develop and regulate cancer cell activities and fate will improve development of novel therapies for both human and veterinary patients using a “One Health” approach.

## Supporting information

S1 Table Clinical diagnostic, treatment, and outcome data. Clinical variables of cats enrolled in this study. DFS, Disease-Free Survival; ST, Survival Time; OHE, ovariohysterectomy; FS, female ovariohysterectomized (spayed); *, ovariohysterectomy performed at time of tumor excision; FU, female unknown OHE status; NA, not able to assess; DHSA, domestic shorthair; DHLA, domestic longhair; RGDL, ragdoll; PERS, Persian.
(DOCX)
