# Supplementary material for: Intratumoral collagen signatures predict clinical outcomes in feline mammary carcinoma
Source: PLoS One. 2020 Aug 10;15(8):e0236516. doi: 10.1371/journal.pone.0236516 (PMC7416937; doi:10.1371/journal.pone.0236516)
Supplement: S1 Table — Clinical variables of cats enrolled in this study. DFS, Disease-Free Survival; ST, Survival Time; OHE, ovariohysterectomy; FS, female ovariohysterectomized (spayed); *, ovariohysterectomy performed at time of tumor excision; FU, female unknown OHE status; NA, not able to assess; DHSA, domestic shorthair; DHLA, domestic longhair; RGDL, ragdoll; PERS, Persian. (DOCX) [file pone.0236516.s001.docx]

**S1 Table. Clinical diagnostic, treatment, and outcome data.**

|  | Signalment at surgery | Largest tumor diameter (cm) | Tumor Type | Grade | Mitotic Count | Lymphatic invasion  Yes (Y) or No (N) | Completeness of excision | Chemo Yes (Y) or No (N) | DFS (Days) | Tumor related event  LN=LN metastasis;  LR=local recurrence; NT = new primary tumor; DM=distant metastasis | ST (Days) | Reason for Censorship |
| --- | --- | --- | --- | --- | --- | --- | --- | --- | --- | --- | --- | --- |
| 1 | 12yo FU DSHA |  | Tubular | III | 68 | N | Clean narrow | N | 65 |  | 65 | Not Censored |
| 2 | 11.5yo FS DSHA | 1 | Tubulopapillary | III | 76 | N | Clean narrow | N | 91 | LR(91) | 155 | Not Censored |
| 3 | 12yo FS Maine Coon | 1.5 | Tubulopapillary | III | 48 | Y | Clean narrow | N | 61 | LR(61)/NT(61) | 68 | Not Censored |
| 4 | 13yo FS DHSA |  | Tubular/ Tubulopapillary | III | 37 | Y | Incomplete | N | 23 | DM(23) | 43 | Not Censored |
| 5 | 10.5yo FS DHSA | 1.5 | Tubular | III | 70 | N | Clean narrow | N | 135 | LR(135)/DM(353) | 355 | Not Censored |
| 6 | 13yo FS DHSA |  | Tubulopapillary | III | 25 | Y | Clean wide | N | 448 | LN(448)/LR(448) | 464 | Not Censored |
| 7 | 12yo FS DHSA | 1 | Tubulopapillary/ Solid | III | 65 | N | Clean narrow | N | 128 | LR(128) | 136 | Not Censored |
| 8 | 15yo FS DHSA |  | Tubulopapillary | III | 44 | Y | Clean narrow | N | 92 | DM(92) | 95 | Not Censored |
| 9 | 17yo FS DHSA | 2 | Tubulopapillary | III | 67 | Y | Incomplete | N | 58 | LR(58)/NT(58) | 71 | Not Censored |
| 10 | 5.5yo FS DHSA | 1 | Tubular | III | 49 | Y | Clean wide | N | 322 | LR(322) | 328 | Not Censored |
| 11 | 12yo FS DHSA | 2 | Tubulopapillary | III | 49 | Y | Clean narrow | Y | 150 | LR(150)/NT(192) | 282 | Not Censored |
| 12 | 12yo FS DHSA |  | Solid | II | 52 | N | Clean narrow | N | 182 | DM(182) | 185 | Not Censored |
| 13 | 15yo FS DLHA | 3 | IDP | I | 13 | N | Clean wide | N | 1137 |  | 1137 | Not Censored |
| 14 | 7yo FU DLHA | 3.5 | Tubular - anaplastic | III | 43 | Y | NA | N | 30 | LR(30) | 36 | Not Censored |
| 15 | 12.5yo FS DHSA | 2 | Tubulopapillary | II | 7 | N | Clean wide | N | 500 | LR(500)/DM(500) | 522 | Not Censored |
| 16 | 12yo FS DHSA | 1 | IDP | I | 7 | N | Clean wide | N | 430 | LR(430) | 854 | Not Censored |
| 17 | 9.5yo FU DHSA | 3 | Cystic - papillary | II | 25 | N | Clean wide | N | 1991 | DM(1991) | 1,991 | Not Censored |
| 18 | 15.5yo FS RGDL | 2 | Tubular/ solid | III | 62 | N | Clean wide | N | 754 |  | 754 | Not Censored |
| 19 | 6yo FS* DHSA |  | Tubular | III | 7 | Y | Clean wide | Y | 414 | LN(414)/LR(590) | 1,070 | Not Censored |
| 20 | 12yo FS DHSA |  | Tubulopapillary - cystic | II | 18 | N | Clean wide | Y | 392 | DM(392) | 514 | Not Censored |
| 21 | 12yo FS RGDL | 1 | Tubulopapillary | III | 64 | N | Clean wide | N | 285 | DM(285) | 285 | Not Censored |
| 22 | 10.5yo FS DHSA | 3 | Solid | III | 88 | N | Clean narrow | N | 158 | NT(158)/DM(158) | 205 | Not Censored |
| 23 | 17yo FS DLHA | 5 | Tubulopapillary | III | 17 | Y | Incomplete | N | 235 |  | 235 | Not Censored |
| 24 | 10yo FS DHSA | 3 | Tubular | III | 49 | Y | Clean narrow | Y | 75 | LR(75)/NT(124)/DM(75) | 192 | Not Censored |
| 25 | 17.5yo FS DHSA |  | Tubulopapillary | III | 22 | Y | Clean wide | N | 97 | NT(97)/DM(97) | 98 | Not Censored |
| 26 | 13yo FS DHSA | 3 | Solid | III | 16 | Y | Clean wide | Y | 203 |  | 203 | Alive |
| 27 | 15yo FS DHLA | 1 | Tubulopapillary | III | 25 | Y | Clean wide | Y | 380 | LR(380) | 477 | Not Censored |
| 28 | 13yo FS DHSA | 3 | Tubular | III | 26 | Y | Clean wide | N | 23 | LR(23)/DM(78) | 78 | Not Censored |
| 29 | 13yo FS PERS | 3 | Tubulopapillary - MP | II | 15 | N | Clean wide | Y | 1381 |  | 1381 | Alive |
| 30 | 11yo FS DHSA | 3 | Solid | II | 36 | N | Clean wide | Y | 1695 | LR(1695) | 1897 | Not Censored |

Clinical variables of cats enrolled in this study. DFS, Disease-Free Survival; ST, Survival Time; OHE, ovariohysterectomy; FS, female ovariohysterectomized (spayed); *, ovariohysterectomy performed at time of tumor excision; FU, female unknown OHE status; NA, not able to assess; DHSA, domestic shorthair; DHLA, domestic longhair; RGDL, ragdoll; PERS, Persian.
